# ﻿Two new combinations of *Middletonia* (Gesneriaceae) in China

**DOI:** 10.3897/phytokeys.261.151963

**Published:** 2025-08-05

**Authors:** Qi-Yang Li, Xin-Xiang Bai, Song-Tao He

**Affiliations:** 1 Forestry College, Guizhou University, CN-5500252 Guiyang, China Guizhou University Guiyang China

**Keywords:** Loxocarpinae, *Middletoniachangjiangensis*, *Middletoniahainanensis*, morphology, *Paraboea*, phylogeny

## Abstract

In 2016, systematic studies of the tribe Loxocarpinae A.DC. supported the establishment of the new genus *Middletonia* C. Puglisi. This study was based on inference and parsimony analyses of a phylogenetic tree derived from the nuclear ITS and plastid *trnL-trnF* regions, constructing the most recent phylogeny that includes the genera *Middletonia* and *Paraboea*. The results showed that the current generic delimitation within the tribe differs from the clades delineated by phylogenetic analysis, and both morphological and molecular evidence support treating the Hainan endemics *Paraboeachangjiangensis* Xing & Z.X.Li and *P.hainanensis* (Chun) Burtt as species of *Middletonia*. Therefore, this paper re-delimits the genera *Middletonia* and *Paraboea*, aiming to establish a more natural classification, and proposes the new combinations *Middletoniachangjiangensis* (F.W.Xing & Z.X.Li) X.X.Bai, **comb. nov.**, and *Middletoniahainanensis* (Chun) X.X.Bai, **comb. nov.**, providing a valuable framework for the development and comparative studies of the molecular systematics of the family Gesneriaceae.

## ﻿Introduction

*Middletonia* C. Puglisi belongs to the subtribe Loxocarpinae A.DC. (1845) of Gesneriaceae. In 2016, Puglisi et al. used molecular data from 68 species of five genera within the subtribe Loxocarpinae to construct a phylogenetic tree. They taxonomically treated *Middletoniaevrardii*, *M.monticola*, and *M.multiflora*, which were originally classified in *Paraboea*, and established the new genus *Middletonia*. They also mentioned that this genus shares similarities in floral morphology with *Paraboea*, which requires further investigation (Puglisi et al. 2016). In the latest taxonomic revision framework, *Middletonia* comprises five species, with their fruit types being twisted or erect (Puglisi and Middleton 2017; GRC 2025).

*Paraboea* (C.B. Clarke) Ridl., established in 1905, also belongs to the subtribe Loxocarpinae of Gesneriaceae (Ridley 1905; Xu et al. 2008). It is the most species-rich genus within this subtribe and has fruit types that are twisted or erect (Weber et al. 2013; GRC 2025). Although *Paraboea* has undergone multiple systematic revisions (Wang et al. 1990, 1998; Li and Wang 2005; Xu et al. 2008; Wei et al. 2010), many taxonomic problems remain unresolved. Thereafter, Guo (2016) conducted a comprehensive study on the genus *Paraboea* in China using molecular systematics combined with morphological traits. The results revealed that *Paraboeahainanensis* (Burtt 1984) and *Paraboeachangjiangensis* (Xing and Li 1993) are significantly distinct from the morphological characteristics of *Paraboea*, with oblique tubular corollas and distinct upper and lower lips. It was proposed that these two species be treated as a new genus, “Huàn Yōng Jù Tái Shǔ” (Guo 2016).

At present, the taxonomic statuses of *Paraboeahainanensis* and *Paraboeachangjiangensis* remain unclear due to limitations in molecular sampling and uncertainty regarding their resource backgrounds. Xu et al. (2017) published three new species of *Paraboea*, using *Middletonia* as an outgroup to construct a phylogenetic tree, but *P.changjiangensis* and *P.hainanensis* were not included in that analysis. Subsequently, Wang et al. (2022) used the chloroplast genomes of 12 *Paraboea* species to construct a phylogenetic tree to verify the monophyly of *Paraboea*, which also did not include *P.changjiangensis* and *P.hainanensis*. However, in the report of the new species *P.zunyiensis*, *P.hainanensis* was found to cluster separately, which does not support the monophyly of *Paraboea* (Deng et al. 2023).

In order to further resolve the complex evolutionary events and developmental relationships, we employed the internal transcribed spacer (ITS) region of nuclear ribosomal DNA (nrDNA) and the *trnL-trnF* intergenic spacer region of chloroplast DNA (cpDNA) to test whether the current classification is consistent with the phylogenetic structure of this group. We aimed to identify robust phylogenetic entities suitable for redefining generic boundaries, address intergeneric and intrageneric issues, and complement the taxonomic treatment with morphological characteristics.

## ﻿Materials and methods

### ﻿Morphological comparisons

In 2023, during a botanical survey in Guizhou Province and Hainan Province, China, the species *Paraboeahainanensis* and *Paraboeachangjiangensis* were found. Subsequently, some living specimens were introduced and cultivated at Guizhou University for further research. We obtained information on *P.hainanensis*, *P.changjiangensis*, and their related species from the Internet, including descriptions in original literature (Chen 1974; Xing and Li 1993) and relevant literature (Brown 1839; Xu et al. 2008), as well as geographic distribution data from the Global Biodiversity Information Facility (GBIF, https://www.gbif.org). In addition, we consulted digital plant specimens collected by E, IBK, PE, NY, GH, and IBSC to examine the type specimens and high-resolution images, as well as other specimens. Finally, we conducted a preliminary taxonomic treatment of the genus by combining existing morphological data and molecular systematic evidence.

### ﻿Genomic DNA extraction, PCR amplification, and sequencing

Species leaf samples collected from the place of origin were quickly dried with silica gel for DNA extraction (Chase and Hills 1991). The nuclear ribosomal internal transcribed spacer (ITS) and chloroplast DNA sequences (*trnL-trnF*) of these samples were amplified by polymerase chain reaction (PCR) using the primers described in Taberlet et al. (1991) and White et al. (1990). All DNA samples were sent to Sangon Biotech Co. Ltd. (Shanghai, China) for sequencing and splicing.

### ﻿Phylogenetic analysis

In the phylogenetic analysis, the ingroup consisted of 33 species from six genera of the Loxocarpinae subtribe, including 26 species of the genus *Paraboea*. Two species from the genus *Petrocodon* in the Didymocarpinae subtribe, *Petrocodonainsliifolius* and *P.viridescens*, were selected as outgroups (Suppl. material 1).

All sequences were compared using MAFFT v.7.5.1.1 (https://mafft.cbrc.jp/alignment/server/) (Katoh and Standley 2013), conserved regions were selected using Gblock, and the substitution saturation index (Iss) of the data matrix was evaluated using DAMBE v5.3.19 (Xia 2013), which showed the Iss < Iss.c, *P* = 0.0000 of the data matrix < 0.05, which is not saturated, can be used to construct phylogenetic trees. Multi-gene syndication was performed using Model Finder software, and polygenes were performed in PAUP*4.0 b10 (Swofford and Sullivan 2003). Thereafter, in Phylosuite v.1.2.3 (Kalyaanamoorthy et al. 2017; Zhang et al. 2020; Xiang et al. 2023), for ML analysis and BI inference. The *trnL-trnF* and ITS sequences were concatenated in series in the Concatenate Sequence module of PhyloSuite v.1.2.3 (Zhang et al. 2020), and the optimal base substitution models for four partitions of the combined dataset were determined in the PartitionFinder2 module using the corrected Akaike Information Criterion (AICc). The best-fitting evolutionary models were GTR+G (*trnL-trnF*) and GTR+I+G (ITS). The ML method employed 1,000 bootstrap replicates to assess the reliability of each node in the phylogenetic tree. The BI rule uses the best substitution model for different segments and independently estimates the Bayesian posterior values of each segment’s parameters, with a random tree as the starting tree, and the initial setting runs for 100,000,000 generations, with one tree reserved for every 10,000 generations. The first 25% of the trees are discarded as burn-in, and the remaining trees are used to generate consensus trees and calculate Bayesian posterior probabilities. Finally, use the iTOL v4 version of the online tool (https://itol.embl.de) to beautify the phylogenetic tree.

## ﻿Results

### ﻿Morphological comparisons

**Figure 1.**
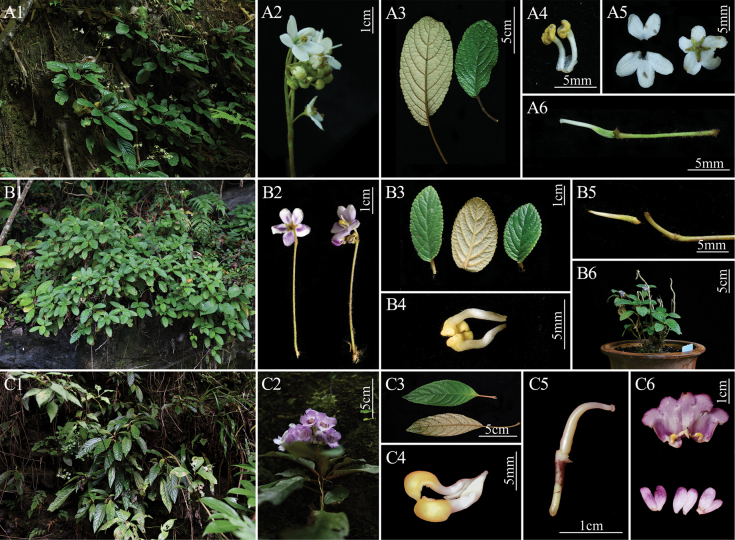
The morphologically and molecularly related species *Middletoniamultiflora*, *Paraboeachangjiangensis*, and *P.sinensis*. A1–6. *M.multiflora*; A1. Habit; A2. Cymes; A3. Petiole and abaxial leaf surfaces; A4. Filaments and anthers; A5. Top view of the expanded corolla tube and calyx; A6. Pistil; B1–6. *P.changjiangensis* B1. Habit; B2. Cymes; B3. Petiole and abaxial leaf surfaces; B4. Filaments and anthers; B5. Calyx; B6. Plants; C1–6. *P.sinensis*; C1. Habit; C2. Cymes; C3. Petiole and abaxial leaf surfaces C4. Filaments and anthers; C5. Pistil; C6. Top view of opened corolla showing the interior surface of the corolla tube, stamens, and staminodes (photographed by Xin-Xiang Bai).

Based on field surveys and specimen examinations, morphological comparisons were conducted between *Paraboeachangjiangensis* and *Paraboeahainanensis* with the type species of *Middletonia* and *Paraboea*, respectively (Table 1). Like *Paraboea*, *Middletonia* presents a matted, interwoven indumentum of long and fine hairs on the abaxial side of the leaf, a flat-faced corolla, and a capsular fruit (Puglisi and Middleton 2017). Combining morphological descriptions from specimens and original literature, the two species can be well distinguished from *Paraboea* based on four morphological characters: corolla, filament, anther, and style morphology, as well as ovary indumentum (Table 1, Fig. 1).

**Table 1.** Comparison among *Middletoniamultiflora*, *Paraboeachangjiangensis*, *P.hainanensis*, and *P.sinensis*.

Characters	*M.multiflora*	*P.changjiangensis*	*P.hainanensis*	*P.sinensis*
Leaf blade shape	oblong or ovate, 7–10 × 4–6 cm, apex rounded-obtuse, base cuneate	elliptic or oblong, 2–7 × 1–3 cm, apex obtuse, base cuneate	narrowly oblanceolate to oblanceolate, 8–18 × 3–6 cm, apex rounded, base gradually attenuate	oblong, oblanceolate or lanceolate, 5.5–25 × 2.4–9 cm, apex acute, base cuneate or broadly cuneate.
Petiole	3–5 cm long, with a matted indumentum	1–2.5 cm long, with a matted indumentum	leaves sessile	3–6 cm long, tomentose brown
Corolla	almost flat-faced	almost flat-faced	almost flat-faced	obliquely campanulate
Anthers	erect	erect	erect	borne at a right angle
Style shape	linear	linear	linear	upper part swollen and saccate, lower part curved and attenuate
Ovary indumentum	grayish-white waxy powder	farinose glandular	some minute pubescence	glabrous

**Figure 2.**
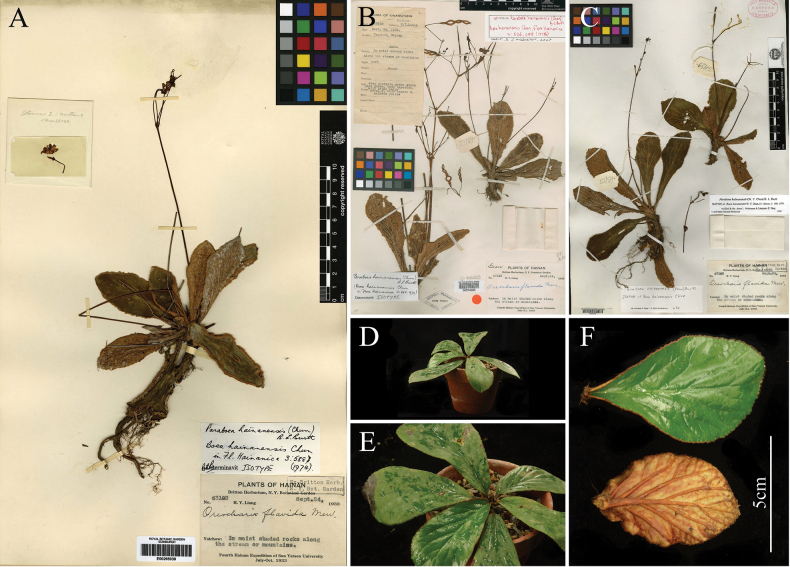
Morphological comparisons of *Paraboeahainanensis*. A. E00067459; B. NY00074065; C. GH00025112; D. Front view of the plant; E. Top view of the plant; F. Leaves (Images D–F were photographed by Qi-Yang Li).

In addition, we compared the collected *Paraboeahainanensis* with the digital specimens (isotypes E00265039, NY00074065, GH00025112) from PPBC and GBIF, as well as the morphological descriptions in relevant literature (Fig. 2, Xu et al. 2008). Sessile leaves are one of the main characteristics of *Paraboeahainanensis*, which are present in all three digital specimens. Moreover, the felt-like indumentum on the lower leaf surface is thick, tightly appressed, and reddish-brown, which is morphologically identical to that of the digital specimens. Furthermore, we carefully compared the leaf shape and size and observed that the leaves of the collected specimens are mostly narrow obovate, rarely narrow elliptic, 2.5–4 times as long as wide, with minute serrations on the margin, which is highly consistent with the images in PPBC and the records in Xu et al. (2008) (Fig. 2).

### ﻿Plastome genome features

**Figure 3.**
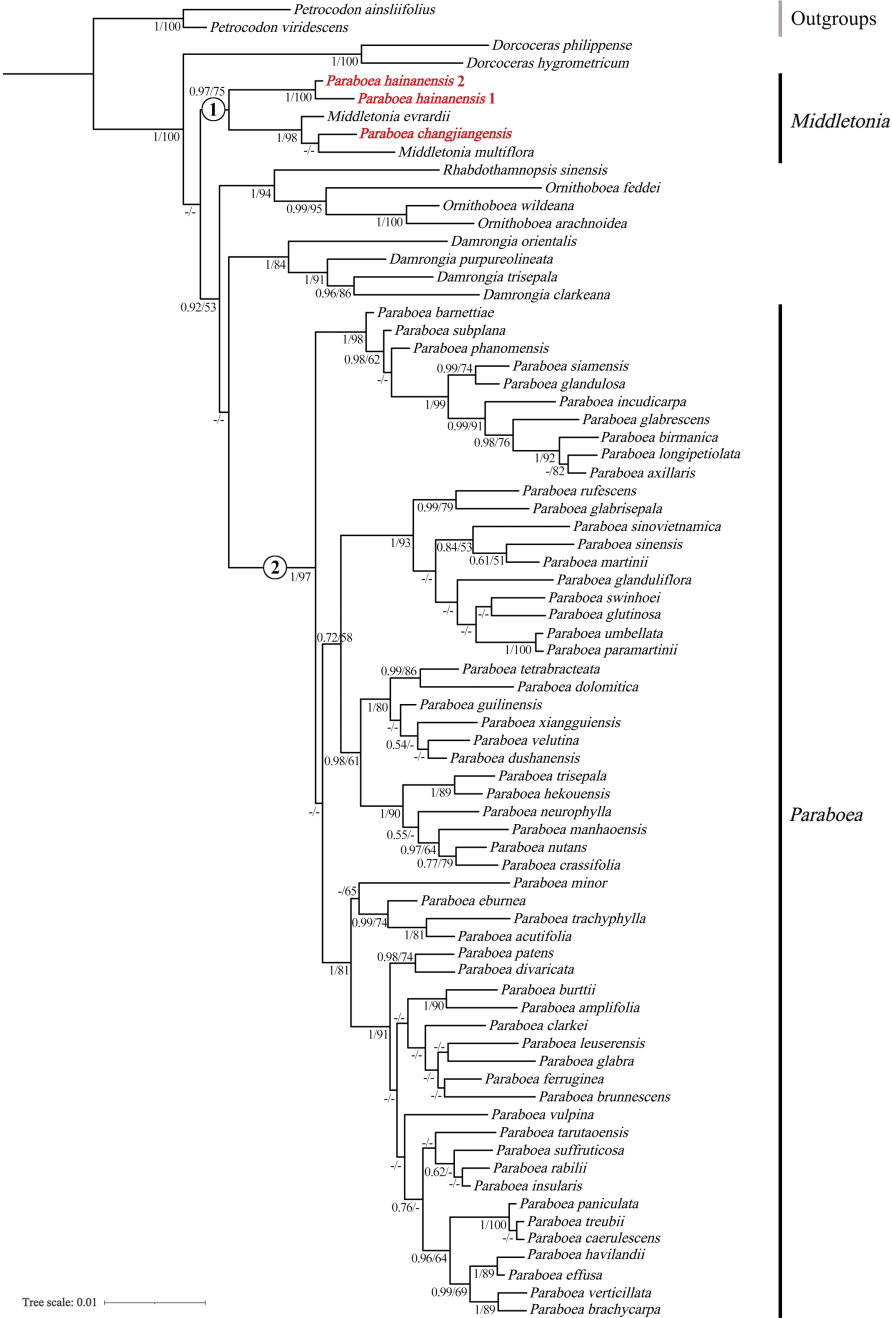
Bayesian tree from analysis of combined ITS and *trnL-F* data. The posterior probabilities (PP) of BI and bootstrap values (BS) of ML are listed at each node. A dash denotes branches with support rates below 50%.

Through combined parsimony and Bayesian inference analyses of ITS and *trnL-F* (Fig. 3), the results showed that in Clade 1, *Paraboeachangjiangensis* was nested within *Middletonia* with high support (posterior probability [PP] = 1.00, bootstrap [BS] = 98%); *Paraboeahainanensis* also formed a strongly supported sister group relationship with *Middletonia* (posterior probability [PP] = 0.97, bootstrap [BS] = 75%). This indicates that classifying these two species in *Paraboea* is incorrect. Additionally, Clade 2 included all species except *Paraboeahainanensis* and *P.changjiangensis*, with high support (posterior probability [PP] = 1.00, bootstrap [BS] = 97%). Therefore, the taxonomic positions of *Paraboeahainanensis* and *P.changjiangensis* are inappropriate, and both should be classified within *Middletonia* (Fig. 3).

### ﻿Taxonomic treatment

Based on the outcomes of phylogenetic and morphological research, both *Paraboeachangjiangensis* and *P.hainanensis* are incorporated into *Middletonia*. The new combinations of *Middletonia* are provided below.

#### ﻿ Middletonia changjiangensis

Taxon classificationPlantaeLamialesGesneriaceae

(Xing & Z.X.Li) X.X.Bai comb. nov.

79B6E1E8-C3AC-561A-871D-02695D3C653A

urn:lsid:ipni.org:names:77366493-1

Fig. 4

≡ Paraboeachangjiangensis Xing & Z.X.Li in *Acta Botanica Yunnanica* 15(2): 121–122, f. 1. 1993. Type: China, Hainan: Changjiang County, Wangxia, 600 m, 25 July 1989, *Z.X.Li & F.W.Xing 5134* (holotype: IBSC!).

##### Distribution and habitat.

Hainan Province: Changjiang County and Dongfang County. The species grows on calcareous formations at an elevation of 600 m.

**Figure 4.**
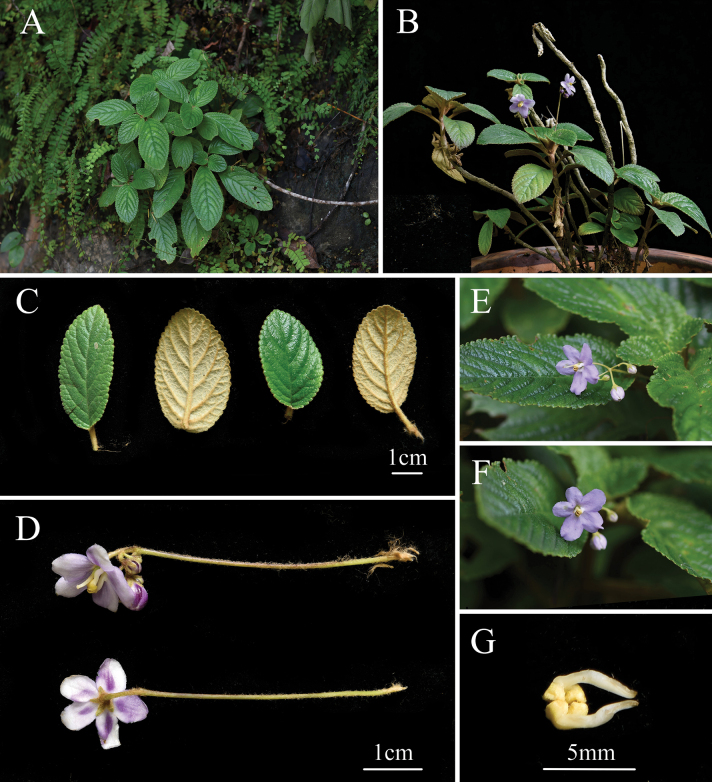
*Middletoniachangjiangensis*. A. Habit; B. Plants; C. Petiole and abaxial leaf surfaces; D. Cymes; E–F. Plant with flowering; G. Filaments and anthers (photographed by Xin-Xiang Bai).

##### Vernacular name.

Chāng Jiāng Fěn Máo Jù Tái (Chinese pronunciation); 昌江粉毛苣苔 (Chinese name).

##### Representative specimens examined.

**China. Hainan Province**: • Changjiang County, 25 Jun 1975, *Guoyuan Fu 264* (IBSC); • Changjiang County, 5 May 1988, *Zexian Li et al.4167* (IBSC).

#### ﻿ Middletonia hainanensis

Taxon classificationPlantaeLamialesGesneriaceae

(Chun) X.X.Bai comb. nov.

B2F5495D-1E0F-5A23-98C7-D5C36CD98DC2

urn:lsid:ipni.org:names:77366494-1

Fig. 2D–F

≡ Boeahainanensis Chun in *Flora Hainanica* 3:526, 588, f. 903. 1974. Type: China, Hainan, Yaichow, on moist shaded rocks along streams in mountains, 18°30'N, 109°08'E, 24 September 1933, *H. Y. Liang 63102* (isotype: E!, NY!, GH!).
≡ Paraboeahainanensis (Chun) Burtt in Notes from the Royal Botanic Garden, Edinb 41(3): 429. 1984.

##### Distribution and habitat.

Hainan Province: Dongfang County. The species grows on moist, shaded rocks along the stream or mountains at an elevation of 800 m. Reported as occurring on acid soil.

##### Vernacular name.

Hǎi Nán Fěn Máo Jù Tái (Chinese pronunciation); 海南粉毛苣苔 (Chinese name).

##### Representative specimens examined.

**China. Hainan Province**: • Ledong County, Aug 1985, *Xinqi Liu 27407* (PE!); • Dongfang County, Aug 1985, *Shaoqing Chen 11251* (IBSC!); • Changjiang County, 0–800 m elev., 25 Jun 1975, Guoyuan Fu 264 (IBSC!); • Changjiang County, 1400 m elev., 20 Aug 2004, *The Kadoorie Project Team of Hong Kong 6632* (PE!); • Hainan, 26 Sep 1933, *Xiangri Liang 63162* (IBK!); • Hainan, 24 Sep 1933, *H. Y. Liang 63102* (E!; NY!).

## ﻿Discussion

In this study, from the perspective of macroscopic morphology, *Paraboeachangjiangensis* and *Paraboeahainanensis* are most closely related to *Middletonia*. Additionally, molecular phylogenetic analyses clarified the phylogenetic relationships between *Paraboea* and *Middletonia*, showing that *P.changjiangensis* is nested within *Middletonia* and *P.hainanensis* forms a strongly supported sister group with *Middletonia*. Future research should focus on resolving the relationships among clades. Additionally, in modern systematics, the integration of molecular and morphological data is indispensable. More molecular markers should be introduced, with priority given to conserved chloroplast region sequences, so as to provide strong support for the backbone topology of the phylogenetic tree. Therefore, we formally rename the two species as *Middletoniachangjiangensis* and *Middletoniahainanensis*, contributing to a more comprehensive phylogenetic framework of Gesneriaceae.

## Supplementary Material

XML Treatment for Middletonia changjiangensis

XML Treatment for Middletonia hainanensis

## ﻿Additional information

### Use of AI

No use of AI was reported.

### Author ORCIDs

Qi-Yang Li https://orcid.org/0009-0006-9336-7718

Xin-Xiang Bai https://orcid.org/0000-0003-2449-6664

Song-Tao He https://orcid.org/0009-0005-5223-7522

### Data availability

All of the data that support the findings of this study are available in the main text or Supplementary Information.

## Supplementary materials

Supplementary material 1 Supplementary information Data type xlsx https://binary.pensoft.net/file/1388359This dataset is made available under the Open Database License (http://opendatacommons.org/licenses/odbl/1.0/). The Open Database License (ODbL) is a license agreement intended to allow users to freely share, modify, and use this Dataset while maintaining this same freedom for others, provided that the original source and author(s) are credited.Qi-Yang Li, Xin-Xiang Bai, Song-Tao He
